# Efficient Secretion and Recombinant Production of a Lactobacillal α-amylase in *Lactiplantibacillus plantarum* WCFS1: Analysis and Comparison of the Secretion Using Different Signal Peptides

**DOI:** 10.3389/fmicb.2021.689413

**Published:** 2021-06-14

**Authors:** Anh-Minh Tran, Kridsada Unban, Apinun Kanpiengjai, Chartchai Khanongnuch, Geir Mathiesen, Dietmar Haltrich, Thu-Ha Nguyen

**Affiliations:** ^1^Food Biotechnology Laboratory, Department of Food Science and Technology, BOKU-University of Natural Resources and Life Sciences, Vienna, Austria; ^2^Department of Biology, Faculty of Basic Sciences, University of Medicine and Pharmacy at Ho Chi Minh City, Ho Chi Minh City, Vietnam; ^3^Division of Biotechnology, Faculty of Agro-Industry, Chiang Mai University, Chiang Mai, Thailand; ^4^Division of Biochemistry and Biochemical Technology, Department of Chemistry, Faculty of Science, Chiang Mai University, Chiang Mai, Thailand; ^5^Research Center of Microbial Diversity and Sustainable Utilization, Faculty of Science, Chiang Mai University, Chiang Mai, Thailand; ^6^Faculty of Chemistry, Biotechnology and Food Science, Norwegian University of Life Sciences (NMBU), Ås, Norway

**Keywords:** *Lactiplantibacillus plantarum*, α-amylase, pSIP expression system, protein secretion, signal peptide, RT-qPCR

## Abstract

Lactic acid bacteria (LAB) have been used as starter cultures and producers of enzymes, antimicrobial peptides or metabolites that contribute to the flavor, texture and safety of food products. *Lactiplantibacillus plantarum*, one of the best-studied LAB, is considered as safe and effective cell factory for food applications. In this study, our aim was to use *L. plantarum* as the producer for high levels of a food-grade lactobacillal α-amylase, which has potential applications in food, fermentation and feed industries. The native form of an α-amylase (AmyL) from *L. plantarum* S21, an amylolytic LAB isolated from Thai fermented rice noodles, was expressed in *L. plantarum* WCFS1 using the pSIP expression system. The secretion of the α-amylase was driven by the native signal peptides of the α-amylases from *L. plantarum* S21 (SP_AmyL) and *Lactobacillus amylovorus* NRRL B-4549 (SP_AmyA), as well as by three Sec-type signal peptides derived from *L. plantarum* WCFS1; Lp_2145, Lp_3050, and Lp_0373. Among the tested signal peptides, Lp_2145 appears to be the best signal peptide giving the highest total and extracellular enzymatic activities of α-amylase AmyL from *L. plantarum* S21, which were 13.1 and 8.1 kU/L of fermentation, respectively. These yields were significantly higher than the expression and secretion in *L. plantarum* WCFS1 using the native signal peptide SP_AmyL, resulting in 6.2- and 5.4-fold increase in total and extracellular activities of AmyL, respectively. In terms of secretion efficiency, Lp_0373 was observed as the most efficient signal peptide among non-cognate signal peptides for the secretion of AmyL. Real-time reverse-transcriptase quantitative PCR (RT-qPCR) was used to estimate the mRNA levels of α-amylase transcript in each recombinant strain. Relative quantification by RT-qPCR indicated that the strain with the Lp_2145 signal peptide-containing construct had the highest mRNA levels and that the exchange of the signal peptide led to a change in the transcript level of the target gene.

## Introduction

Amylases are common enzymes that have been applied widely in many industrial processes owing to their catalytic ability in starch hydrolysis (El-fallal et al., 2012). They have been employed in industries such as food, fermentation, textile, paper, pharmaceutical, and fine chemicals industries (Gupta et al., 2003; Kandra, 2003; El-fallal et al., 2012). Amylases are among the earliest industrial enzymes and their role is irreplaceable in industries requiring starch conversion. One of the most important forms of amylases is the α-amylase (1,4-α-D-glucan glucanohydrolase, EC. 3.2.1.1) (Sindhu et al., 2017). This enzyme hydrolyzes internal α-D-(1–4) glycosidic bonds of starch into shorter oligosaccharides consisting of three or more glucose units (Kandra, 2003). α-Amylases can be obtained from plants, animals, and various species of microorganisms. However, microbial α-amylases attract more attention because their stability surpasses that from other sources. In addition, microbial production of α-amylases has a number of advantages such as low production cost, ease of scaling-up, and desired characteristics of the enzymes can be acquired by genetic modifications (Gupta et al., 2003; de Souza and de Oliveira Magalhães, 2010).

*Lactiplantibacillus plantarum* as it had been renamed recently (Zheng et al., 2020) is one LAB species that can exhibit amylolytic activity. These amylolytic LAB express α-amylase as a main extracellular enzyme during bioconversion of starch to lactic acid in a single step, which is an attractive property to reduce lactic acid production expenses (Kanpiengjai et al., 2015a; Padmavathi et al., 2018). *L. plantarum* is also of interest because it is considered as safe (GRAS; generally recognized as safe), thus can be vastly applied in food and pharmaceutical industries. Recently, Kanpiengjai et al. (2014, 2015a) isolated the *L. plantarum* strain S21 from Thai fermented rice noodles and studied the enzymatic properties of an α-amylase (AmyL) produced by this strain. AmyL is a monomeric enzyme with 910 amino acids including the signal peptide, Ca^2+^ independent, and the optimum of its activity was found at pH 5.0 and 45°C. Despite of sharing more than 96% amino acid sequence identity with the α-amylases from *L. plantarum* A6, *L. manihotivorans* LMG18010, and *L. amylovorus* NRRL B-4540, AmyL possesses superior stability over a broad range of pH. It retained 80–95% of its initial activity over the pH range of 4–8 at 37°C for 12 days and was catalytically active after 30 days under the same conditions with 70–75% residual activity. The pattern of its hydrolysis products obtained after the conversion of polymeric substrates such as starch, amylose, amylopectin, and glycogen indicated that this enzyme acts as both liquefying and saccharifying enzyme, and this pattern was also highly different from that of other amylolytic lactic acid bacteria (Kanpiengjai et al., 2015a).

*Lactiplantibacillus plantarum* WCFS1 has been exploited as a host for intracellular expression, secretion and cell-surface display of heterologous proteins (Mathiesen et al., 2004; Halbmayr et al., 2008; Michon et al., 2016; Nguyen et al., 2016; Sak-Ubol et al., 2016; Stern et al., 2018) using the pSIP expression systems (Sørvig et al., 2003, 2005; Mathiesen et al., 2008). Kanpiengjai et al. (2015b) successfully used the pSIP409 expression system to produce AmyL in *L. plantarum* WCFS1 using its native signal peptide sequence, which resulted in 91% secretion efficiency of the AmyL and ∼ 2.1 kU/L of fermentation medium in the extracellular fraction. In *L. plantarum* most secreted proteins are translocated with the Sec secretion machinery and the Twin-arginine translocation (Tat) pathway is absent (Kleerebezem et al., 2003; Liu et al., 2015; Zhang et al., 2018; Huang et al., 2020). Therefore, secretory pre-proteins are translocated across the cell membrane as unfolded polypeptides, and during the translocation a signal peptidase type I catalyzes the cleavage of the N-terminal signal peptide sequences from the preproteins before the proteins are released into the medium (van Roosmalen et al., 2004; Watanabe et al., 2009; Anné et al., 2017). This feature can be exploited to improve heterologous protein secretion in *L. plantarum*, e.g., replacing the signal peptide in a protein can facilitate increased secretion efficiency. Mathiesen et al. (2009) constructed a library of 76 Sec-type signal peptides derived from *L. plantarum* WCFS1. Using a staphylococcal nuclease (NucA) and the truncated form of a α-amylase (AmyA) derived from *L. amylovorus* as reporter proteins, they identified several signal peptides that resulted in efficient secretion of the reported proteins. However, the performance of the signal peptides may vary depending on the protein that is secreted (Mathiesen et al., 2009; Peng et al., 2019).

Besides the effects of signal peptides on protein secretion in context of protein translocation, mRNA stability, one of the main factors affecting the amount of mRNA in the cell, significantly influences the levels of protein synthesis (Carrier and Keasling, 1997). mRNA levels of a gene of interest can be determined using reverse transcription quantification real-time PCR (RT-qPCR). This powerful tool for the detection and quantification of mRNA has been widely used because of its high sensitivity, reproducibility and wide dynamic quantification range (Pfaffl et al., 2002; VanGuilder et al., 2008). This approach requires a suitable set of housekeeping or reference genes as internal controls for normalizing data of target gene expression (Vandesompele et al., 2002). To gain a reliable and accurate measurement, mRNA synthesis of housekeeping genes is required to be stable under certain experimental conditions or lacking significant regulation (Vandesompele et al., 2002; Pfaffl et al., 2004). Many studies have proved that the housekeeping genes are regulated and vary through different conditions though, and thus validation of housekeeping genes for normalization is recommended and usage of multiple reference genes is necessary for reliable measurements (Pfaffl, 2001; Vandesompele et al., 2002).

In this present study, we compared different signal peptides for the expression of a lactobacillal α-amylase using *L. plantarum* WCFS1 as the host, aiming at both efficient secretion and high production levels of the α-amylase AmyL from *L. plantarum* S21. We expressed the native form of AmyL using the pSIP401 expression system in combination with five different signal peptides including Lp_2145, Lp_3050, and Lp_0373, which are the Sec-type signal peptides derived from *L. plantarum* WCFS1, and the native signal peptides, SP_AmyL and SP_AmyA, of the α-amylases from *L. plantarum* S21 and *L. amylovorus* NRRL B-4549, respectively. The expression and secretion of AmyL was compared with the truncated form of the α-amylase from *L. amylovorus* NRRL B-4549 (AmyA). Finally, the correlation between secretion and transcript levels in recombinant strains containing different signal peptide sequences was investigated.

## Materials and Methods

### Bacterial Strains and Growth Conditions

*Lactiplantibacillus plantarum* WCSF1, isolated from human saliva as described by Kleerebezem et al. (2003), was originally obtained from NIZO Food Research (Ede, Netherlands) and maintained in the culture collection of the Norwegian University of Life Sciences (NMBU), Ås, Norway. *L. plantarum* WCSF1 was used as an expression host for the vectors harboring α-amylase genes. *L. plantarum* strains were grown anaerobically in deMan, Rogosa, and Sharpe (MRS) broth (Carl Roth, Karlsruhe, Germany) at 37°C without agitation. All solid media were prepared with the supplementation of 1.5% (w/v) agar. *Escherichia coli* NEB5α (New England Biolab, Frankfurt am Main, Germany) was used in the transformation involving subcloning of DNA fragments and grown in Luria-Bertani (LB) broth at 37°C with agitation at 180 rpm. To maintain the plasmids, erythromycin was added into the cultivation media to final concentrations of 200 μg/mL for *E. coli* and 5 μg/mL for *L. plantarum*.

### DNA Manipulation and Transformation

Plasmids were isolated from *E. coli* NEB5α using the Monarch plasmid miniprep kit (New England Biolabs). PCR products and the digested fragments were purified using the Monarch DNA Gel extraction kit (New England Biolabs) and the DNA concentration was estimated by Nanodrop 2000 (Thermo Fisher Scientific, Waltham, MA, United States). DNA amplifications were performed using Q5^®^ High-Fidelity DNA Polymerase (New England Biolabs) and the primers are listed in the Supplementary Table 1. Recombinant plasmids were constructed using restriction enzymes and T4 DNA ligase (New England Biolabs) and then transformed into *E. coli* NEB5α electrocompetent cells. Sequences of PCR generated fragments were verified by DNA sequencing performed by a commercial provider (Microsynth, Vienna, Austria). The constructed plasmids were transformed into *L. plantarum* WCFS1 electrocompetent cells following a protocol described previously (Aukrust and Blom, 1992).

### Plasmid Construction

**FIGURE 1:**
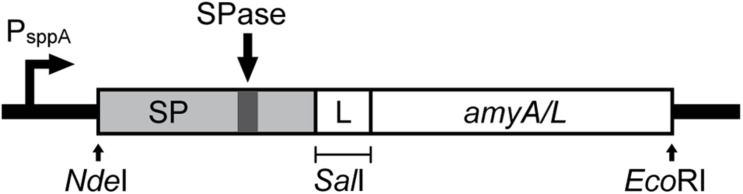
Schematic overview of the expression cassette for the secretory production of α-amylases. The non-native signal peptides (SP) were translationally fused to the *amyA* or *amyL* with the linker GTCGAC, which is a *Sal*I site, located after 2 codons downstream of the signal peptide cleavage site. This expression cassette was inserted in the pSIP401 plasmid.

Figure 1 shows a schematic overview for the construction of the expression cassette for the secretion of the α-amylases, which are AmyL (Accession No. KJ440080.1) from *L. plantarum* S21 and AmyA (Accession No. EF419426.1) from *L. amylovorus* NRRL-B4549, with different signal peptides (SPs). The signal peptides Lp_2145, Lp_3050, Lp_0373, and SP_AmyA used in this study were taken from the plasmids pLp_2145s_AmyA, pLp_3050s_AmyA, pLp_0373s_AmyA, and pLp_spAmyA_AmyA (Supplementary Table 2), respectively, which are derivatives of the pSIP401 vector that have been previously constructed for the expression and secretion of AmyA from *L. amylovorus* NRRL-B4549 (Mathiesen et al., 2008). The signal peptide SP_AmyL is the native signal peptide of AmyL from *L. plantarum* S21, which was previously constructed with its mature gene *amyL* in the plasmid pLp_AmyL7 (Supplementary Table 2; Kanpiengjai et al., 2015b). The sequences of the signal peptides used in this study are listed in Supplementary Table 3.

For construction of the expression plasmids for secretion of AmyL from *L. plantarum* S21, the *amyL* gene fragment, which was PCR generated using the template pLp_AmyL7 (Supplementary Table 2; Kanpiengjai et al., 2015b) and the primer pair AmyL_*Sal*I_Fw and AmyL_*Eco*RI_Rv (Supplementary Table 1), was ligated into the *Sal*I/*Eco*RI-digested vectors pLp_2145s_AmyA, pLp_3050s_AmyA, and pLp_0373_AmyA yielding the plasmids pLp_2145s_AmyL, pLp_3050s_AmyL, and pLp_0373_AmyL, respectively (Figure 1).

For construction of the plasmid pLp_spAmyA_AmyL, firstly the pSIP401 vector fragment with *Sal*I/*Eco*RI restriction sites was PCR-generated using pLp_spAmyA_AmyA as a template and the primer pair 401_spAmyA_*Eco*RI_Fw and 401_spAmyA_*Sal*I_Rv (Supplementary Table 1). A *Sal*I site was introduced at the C-terminus of the signal peptide of AmyA (SP_AmyA) after 2 codons downstream of the SP cleavage site, which was determined using SignalP-5.0^1^ (Armenteros et al., 2019). It serves as a linker between the SP and the target gene as described previously (Mathiesen et al., 2008). The resulting pSIP401 PCR fragment (∼5729 bp) containing the native signal peptide of AmyA (SP_AmyA) was then digested with *Sal*I/*Eco*RI before being ligated with the *Sal*I/*Eco*RI-digested *amyL* gene fragment, yielding the plasmid pLp_spAmyA_AmyL.

### Gene Expression in *L. plantarum* and Sample Collection

The expression plasmids were constructed in *E. coli* NEB5α before electroporation into *L. plantarum* WCFS1 competent cells, and transformants were selected on MRS agar plates containing 5 μg/mL erythromycin. Overnight cultures of *L. plantarum* were diluted in 200 mL of MRS broth containing 5 μg/mL erythromycin to a final optical density (OD) at 600 nm of ∼ 0.1 and incubated at 37°C. When an OD_600_ reached 0.3, inducing peptide IP-673 (Eijsink et al., 1996) was added into the cultures to a final concentration of 25 ng/mL. The growth of *L. plantarum* strains was monitored and samples of 5 or 10 mL of fermentation broth were collected at time intervals. Pellets and supernatants for protein quantification and enzymatic assays were separated by centrifugation at 4000 × *g* for 15 min at 4°C and stored at −20°C. The pellets were washed twice with 0.1 M sodium phosphate buffer pH 6.5 prior to storage. To collect intracellular proteins, the pellets were disrupted on ice by sonication (Bandelin Sonopuls HD60, Bandelin, Berlin, Germany). For RNA extraction, a volume of fermentation broth with biomass equivalent to OD_600_ of 2.0 was collected and the cells were separated using centrifugation at 5000 × *g* for 5 min at 4°C, followed by treatment with 2 × the volume of RNA Protect Reagent (QIAGEN, Hilden, Germany). The treated cells were then stored at −80°C until usage.

### SDS-PAGE Analysis and α-amylase Activity Assay

Protein concentrations were measured by Bradford assay (Bradford, 1976) using bovine serum albumin (BSA, Bradford reagent 5 ×, Bio-Rad, Hercules, CA, United States) as standard. The proteins from supernatant and cell lysate samples were concentrated and exchanged to 0.1 M sodium phosphate buffer pH 6.5 using Amicon Ultra 0.5 mL Centrifugal filters (Sigma-Aldrich, Merck KGaA, Darmstadt, Germany) with 10 kDa cut-off prior to SDS-PAGE. The protein bands were visualized using Bio-Safe Coomassie Brilliant Blue G-250 (Bio-Rad, Hercules, CA, United States).

α-Amylase activity was measured using the DNS method (3,5-dinitrosalicylic acid, Sigma-Aldrich, St. Louis, MI, United States) as described previously (Kanpiengjai et al., 2015a) using D-glucose as standard. One unit of amylase activity was defined as the amount of enzyme releasing 1 μmol of reducing sugars (or reducing end equivalents) per minute under the given conditions.

### Total RNA Extraction and cDNA Synthesis

The cells were thawed on ice and mixed with 110 μL of Tris-EDTA buffer pH 8.0 containing 25 mg/mL lysozyme and 2 mg/mL proteinase K. The reaction mixture was incubated at 25°C for 45 min with agitation at 900 rpm. Total RNA was extracted from the lysate using the peqGOLD Bacterial RNA Kit (VWR International GmbH, Darmstadt, Germany) followed by the removal of genomic DNA using the DNase Max Kit (QIAGEN, Hilden, Germany). RNA quantification and qualification were performed using Nanodrop 2000 (Thermo Fisher Scientific, Waltham, MA, United States). Genomic DNA residues in the RNA samples were clarified using both conventional PCR and real-time PCR (RT-qPCR). All RNA samples were diluted to the same concentration before an input amount of 400 ng of total RNA per 20 μL of reaction mixture was converted into cDNA using iScript^TM^ cDNA Synthesis Kit (Bio-Rad, Hercules, CA, United States) following the manufacturer’s protocol. The cDNA samples were diluted 4- and 100-fold in deionized water prior to dilution series preparation and expression level measurements, respectively. All samples were prepared independently in at least two replicates.

### Selection of Candidate Reference Genes and Real-Time PCR Primer Design

Nine housekeeping genes, *gmk*, *fusA*, *gyrA*, *recA*, *gapB*, *rpoD*, *rho*, *rpoB*, and *ldhD* (Table 1), were selected based on several published reports (Marco et al., 2007; Fiocco et al., 2008; Duary et al., 2010; Wu et al., 2016; Lin et al., 2018). Real-time PCR (RT-qPCR) primers were designed using Primer3 (Koressaar and Remm, 2007; Untergasser et al., 2012)^2^ with their amplicon lengths and annealing temperature set in the range of 70–120 bp and 60°C, respectively. To avoid unexpected amplifications, each primer sequence was aligned against the genomic DNA sequence of *L. plantarum* strain WCFS1 (GenBank Accession No. AL935263.2) using BLAST tool^3^. Potential formation of secondary structures of amplicons generated by each primer pair during PCR was predicted using UNAFold (Markham and Zuker, 2008)^4^ while primer dimerization was checked using NetPrimer (Premier Biosoft International)^5^. All primers were synthesized by a commercial provider (Microsynth, Vienna, Austria). The specificity of the primers was verified by running the RT-qPCR products on 1.5% agarose gel and melt curve analysis. The primer pairs of each target resulting in a single band with expected size on the agarose gel and a single peak in melt curve analysis were selected for the primer concentration optimization step. To obtain the best combination of primer final concentrations for each target, different final concentrations of the forward and reverse primers (300, 400, and 500 nM) in the reaction mixtures were combined and tested with qPCR. The combination giving the lowest Ct value was selected for all subsequent qPCR assays.

**TABLE 1:** Target and housekeeping genes used in this study.

Gene name	Gene function	Accession number/Locus tag	References
*amyL*	*L. plantarum* strain S21 α-amylase	KJ440080.1	Kanpiengjai et al. (2014)
*amyA*	*L. amylovorus* NRRL-B45 α-amylase	EF419426.1	Mathiesen et al. (2008)
*fusA*	Protein translation elongation factor G	AL935263.2/lp_1027	
*gapB*	Glyceraldehyde-3-phosphate dehydrogenase B	AL935263.2/lp_0789	
*gmk1*	Guanylate kinase	AL935263.2/lp_1612	
*gyrA*	DNA replication, DNA gyrase subunit A	AL935263.2/lp_0007	
*ldhD*	D-lactate dehydrogenase	AL935263.2/lp_2057	
*recA*	Recombinase A	AL935263.2/lp_2301	
*rho*	Transcription terminator factor Rho	AL935263.2/lp_0511	
*rpoB*	DNA-directed RNA polymerase subunit beta	AL935263.2/lp_1021	
*rpoD*	RNA polymerase, sigma 70 (sigma D) factor	AL935263.2/lp_1962	

### Relative Quantitative Real-Time PCR Assay

The primers were validated before being applied to RT-qPCR assays with regards to their specificity, the specific melting temperature (T_*m*_) of amplicons, the optimum concentration of each primer at annealing temperature of 60°C followed by amplification efficiency (E%) estimation. The validation data are presented in Table 2. The primer *amy* was designed based on the consensus sequence of the amylase genes from *L. plantarum* S21 and *L. amylovorus* NRRL B-4549. Quantitative Real-time PCR assays (qPCR) were performed in 96-well plates on the MyiQ^TM^ Single-Color Real-Time PCR Detection System (Bio-Rad, Hercules, CA, United States). Each reaction was prepared in a 10 μL mixture containing 5 μL of iTaq^TM^ Universal SYBR^®^ Green Supermix (Bio-Rad, Hercules, CA, United States), 1 μL of each primer with designated final concentration (Table 2), and 3 μL of diluted cDNA. Thermal conditions were as follows: 95°C for 30 s, 40 cycles at 95°C for 15 s, and then at 60°C for 25 s with fluorescence measurement, and 61 cycles of melt curve profiling in which the temperature was increased to 95°C at a rate of 0.05°C s^–1^. All qPCR reactions were performed in triplicates. Non-template control for each target was included. Amplification efficiency (E) of each primer pair was estimated using a 5-fold dilution series of cDNA pool, where E = 10^–1/slope^. Primer pairs giving E (%) in a range of 90–110% and the coefficient of determination *R*^2^ ≥ 0.99 were selected. Obtained E% values were applied on all subsequent analyses.

**TABLE 2:** Oligonucleotide primers used in this study.

Gene name		Primer sequence (5′→3′)	Concentration/reaction (nM)	Amplicon size (bp)	Amplicon Tm (°C)	PCR efficiency (%)
*amy*	Fw	AGTAACTTGGGTCGAATCGCAT	400	99	76	94.0
	Rv	GCAACAACAGCCCAGCCTAAT	500			
*fusA*	Fw	AGACCACGACTACTGAACGG	400	101	78	94.4
	Rv	TTCTTGAGCCATCCAGTCCA	500			
*gapB*	Fw	GTCGTTTAGCATTCCGTCGT	400	98	76.5	94.4
	Rv	AGCCAACAATGCAGGTGAAG	400			
*gmk*	Fw	GGCGAAGTAGATGGCAAGGA	500	116	78	97.0
	Rv	GGCGTCCCATAGTAATTGTCAAC	400			
*gyrA*	Fw	GCAGTCTTACCAGCACGTTTC	500	85	79	97.0
	Rv	GTGGCGGAATGTTTGTTGTCAT	500			
*ldhD*	Fw	CGTCCAAGTTATCAACACCAACG	400	100	76	97.0
	Rv	TTGAACAAGTTAGCCGACGAAG	400			
*recA*	Fw	CAGACGTTTCTTCACCAGTT	400	95	79	92.3
	Rv	GAAGTATTTGGACGAGCATC	500			
*rho*	Fw	AGCGGTCAATCAAGGGAGAA	500	89	79	92.0
	Rv	ACGTTCAAGCACCAATTCCG	400			
*rpoB*	Fw	GCTCGTTCAATCGGACCTTA	500	98	80	92.8
	Rv	GCCCAAACTTCCATTTCACC	400			
*rpoD*	Fw	GCCAGCGTCACTAAGTTCCT	500	96	77.5	97.5
	Rv	GCTGGGATTAGTGTTGTTGATGA	500			

### Determination of Reference Genes During the Exponential Growth Phase of *L. plantarum* WCFS1

Wild-type *L. plantarum* WCFS1 was grown in 200 mL of MRS broth at 37°C for 24 h. Cells were then collected at four time points selected from the exponential growth phase and their RNAs were extracted for RT-qPCR. The qPCR results for candidate reference genes were evaluated using GeNorm (Vandesompele et al., 2002), BestKeeper (Pfaffl et al., 2004), and NormFinder (Andersen et al., 2004).

### Relative Quantification of Transcription Levels of α-amylase Genes

Cells of recombinant *L. plantarum* WCFS1 strains harboring the expression plasmids with different SPs were collected at 0, 3, 6, and 12 h after induction with IP-673 for RNA isolation. Transcription levels of α-amylase genes from the samples were calculated using the REST2009 software based on the comparative method (2^–ΔΔCt^) (Pfaffl et al., 2002) along with three selected reference genes. The strain *L. plantarum* S21 served as control for all measurements. The difference in transcript levels between the target genes and the control was statistically evaluated by REST 2009, so called randomization test method (Pfaffl et al., 2002), and a *p*-value < 0.05 is considered significant.

## Results

### Expression of α-amylases in Recombinant *L. plantarum* WCFS1

The cultivations of the wild-type and the recombinant *L. plantarum* WCFS1 strains harboring AmyL and AmyA expression plasmids were performed at 37°C in MRS medium. All cultures reached OD_600_ of around 8.0 after 18–24 h of cultivation, except the strains containing the constructs with the signal peptide Lp_3050, which had slower growth rates (Supplementary Figure 1).

SDS-PAGE analysis of the supernatant and cell lysates showed that all recombinant bacteria produced and secreted the amylases, AmyL (∼95 kDa) (Supplementary Figure 2A) and AmyA (∼49 kDa) (Supplementary Figure 2B). The intensity of the target bands also indicated that the proteins were produced at different levels given that the same amounts of proteins were applied to the gels. Constructs containing the signal peptides Lp_2145, Lp_0373, and Lp_AmyA showed notably higher recombinant protein yields compared to the strains with the signal peptides Lp_3050 (for both AmyL and AmyA secretion) and SP_AmyL (for AmyL secretion) as judged by SDS-PAGE.

**FIGURE 2:**
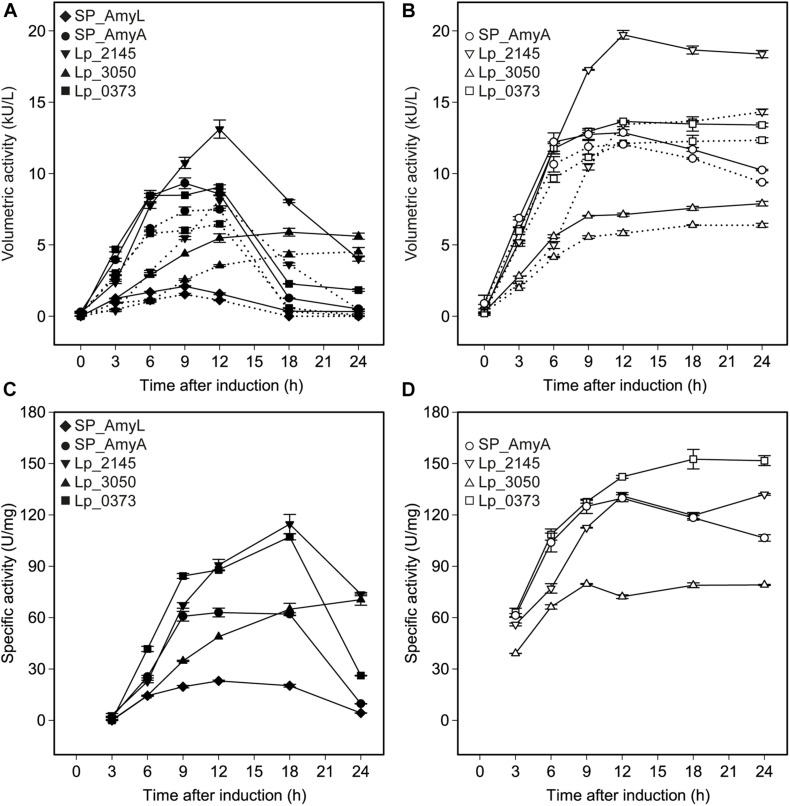
Volumetric and specific α-amylase activities produced by recombinant *L. plantarum* WCFS1 harboring various expression plasmids of AmyL [**(A,C)**, respectively] and AmyA [**(B,D)**, respectively]. The solid lines and the dotted lines in panels **(A,B)** represent total volumetric activities and extracellular activities, respectively. Values given are the average values from at least two independent experiments and the error bars indicate the standard deviation.

**FIGURE 3:**
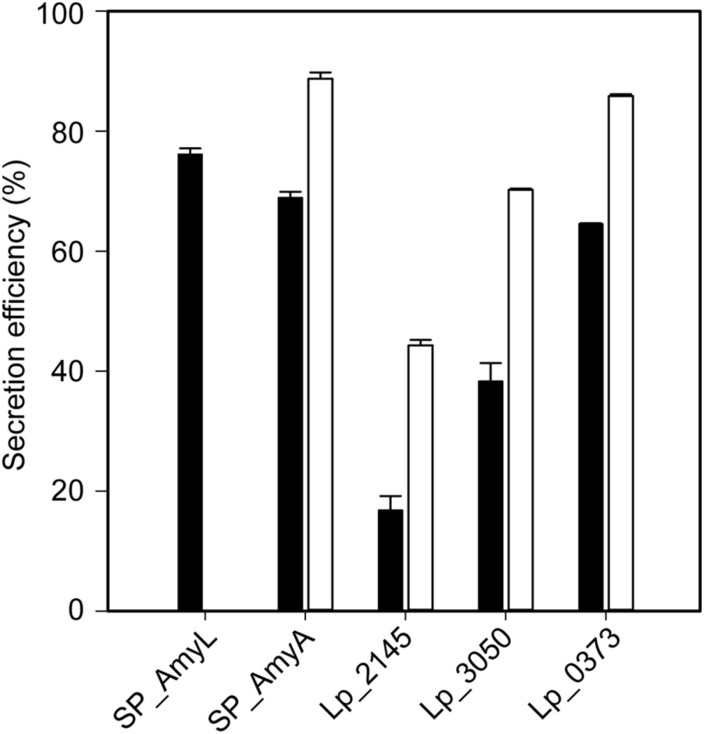
Secretion efficiencies of recombinant *L. plantarum* WCFS1 harboring various expression plasmids of AmyL (black bars) and AmyA (white bars) at 3 h after induction. Values given are the average values from at least two independent experiments and the error bars indicate the standard deviation.

The successful expression of α-amylase was confirmed by enzymatic activity assays. The extracellular volumetric amylase activities of strains containing AmyA secretion plasmids were higher than those of the strains containing AmyL secretion plasmids with respective signal peptides (Figures 2A,B). Notably, AmyA appeared to be stable while AmyL activities dropped quickly after 12 h. Looking at the secretion of AmyL, constructs with the Lp_2145 signal peptide gave the highest extracellular AmyL activity of ∼8.1 kU/L of culture medium with a specific activity of 90 U/mg protein after 12 h of cultivation and OD_600_ of around 5.0, followed by constructs with the signal peptides SP_AmyA and Lp_0373, showing 7.5 and 6.5 kU/L with specific activities of 60 and 90 U/mg, respectively, also after 12 h of cultivation (Figures 2A,C). The lowest production was observed with the construct containing AmyL’s native signal peptide (SP_AmyL), of which the highest extracellular activity was determined to be 1.5 kU/L with 23 U/mg specific activity (Figures 2A,C). For AmyA secretion, constructs with the Lp_2145 signal peptide also showed the highest extracellular AmyA activity reaching ∼13.5 kU/L with a specific activity of 130 U/mg after 12 h of cultivation at OD_600_ of 4.7, followed by constructs with the signal peptide Lp_0373 with ∼12 kU/L and specific activity of 140 U/mg at the same time point (Figures 2B,D). Highest specific activities of both AmyL and AmyA were obtained with constructs using the signal peptides Lp_2145 and Lp_0373 (Figures 2C,D). The two constructs with the signal peptide SP_AmyA reached the highest extracellular activities of both AmyL (7.4 kU/L) and AmyA (12.0 kU/L) at around OD_600_ 6.0 after 9 h of cultivation, which was earlier than the constructs with other signal peptides. No extracellular amylase activity was detected for the wild-type *L. plantarum* WCFS1 while the non-induced strain containing the plasmid pLp_spAmyA_AmyL displayed leaky expression with ∼100 and ∼983 U/L as the maximal extracellular and intracellular AmyL activity, respectively (data not shown). In terms of total α-amylase volumetric activity, constructs with the Lp_2145 signal peptide also showed the highest activities (∼13.1 and ∼19.7 kU/L for AmyA). However, the secretion efficiencies of these strains were lowest compared with the other strains. The most efficient secretion was observed with the strains having the cognate signal peptides SP_AmyA and SP_AmyL (Figure 3). Among the tested heterologous signal peptides, Lp_0373 and Lp_2145 were observed to be the most and the least efficient, respectively, for the secretion of both AmyL and AmyA.

### Reference Genes During the Exponential Growth Phase of *L. plantarum* WCFS1

**FIGURE 4:**
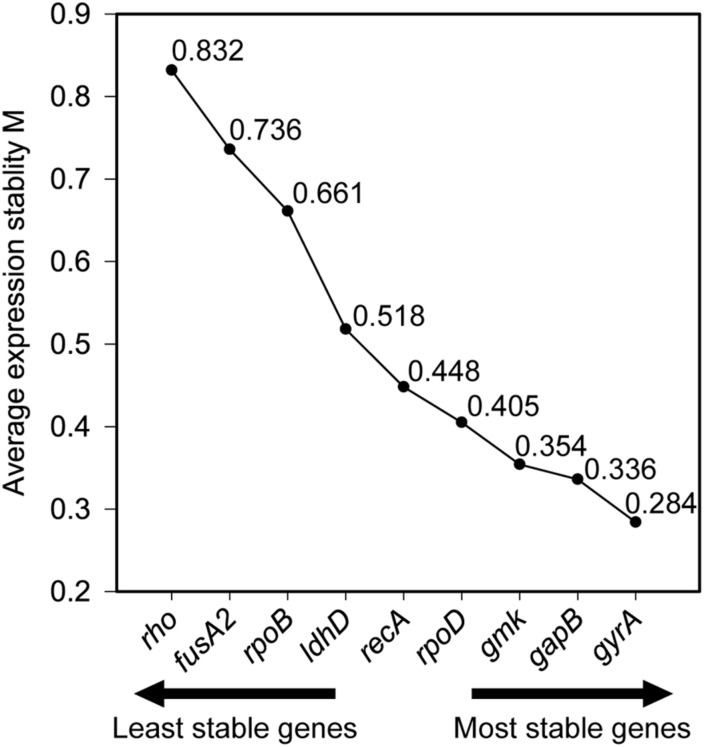
Average expression stability of reference genes calculated using geNorm.

**FIGURE 5:**
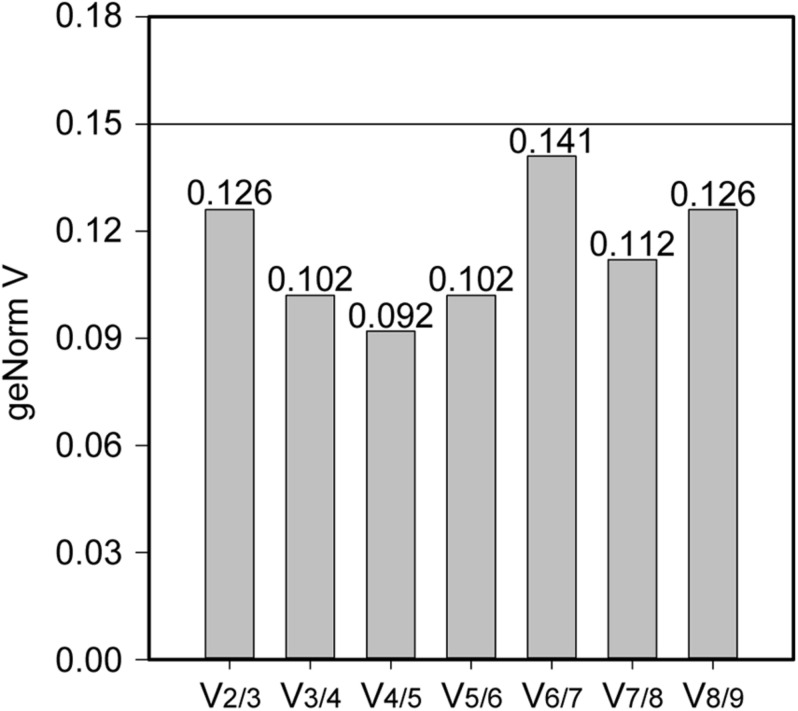
The pair-wise variation generated by geNorm to determine the optimal number of reference genes for accurate measurement of mRNA levels using RT-qPCR. A *V*-value lower than the standard cut-off of 0.15 is considered acceptable.

Stability of the expression of the housekeeping genes during the change of acidic condition and carbon source depletion was evaluated. Particularly, the mRNA samples of *L. plantarum* WCFS1 were collected at the time points at 3, 6, 9, and 12 h after induction and used for RT-qPCR assays. The RT-qPCR results of all nine housekeeping genes were initially analyzed using GeNorm to determine the average expression stability value (M) of each gene. The genes with the lower M values, which were *gyrA*, *gmk*, *gapB*, and *ldhD*, belonged to the group of stable genes (Figure 4). The same analysis was conducted with BestKeeper and NormFinder. BestKeeper determined that *recA*, *rpoD*, *gmk*, and *gyrA* were stable genes according to their standard deviations while NormFinder selected *ldhD*, *gmk*, *gyrA*, and *gapB* based on the stability values. The geometric mean of the results given by these three tools of all nine housekeeping genes was calculated and ranked from the lowest to highest, with the most stable genes on top. Pair-wise variation analysis of nine candidate housekeeping genes using GeNorm showed that all geNorm *V* values were lower than the 0.15 cut-off value, thus the usage of the two most stable genes, namely *gmk* and *gyrA*, would be sufficient for normalization of the target gene (Figure 5). However, at least three reference genes were recommended for more accurate normalization if there are small expression differences, e.g., 2- to 3-fold (Vandesompele et al., 2002). Therefore, *gmk*, *gyrA*, and *gapB* were selected as the reference genes.

### Relative Expression of α-amylase Genes

**FIGURE 6:**
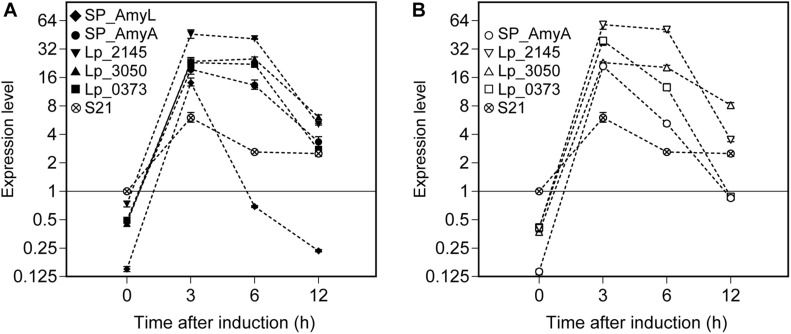
Expression levels of **(A)** *L. plantarum* S21 α-amylase (*amyL*) and **(B)** *L. amylovorus* NRRL B-4549 truncated α-amylase (*amyA*) in recombinant *L. plantarum* WCFS1 strains harboring various secretion plasmids calculated using REST 2009. A cDNA sample of *L. plantarum* S21 at 0 h was used as control, and its expression level was taken as 1. Values given are the average values from at least two independent experiments. The error bars indicate 95% confidence intervals (CI) calculated by REST 2009.

Relative expression of the α-amylase gene constructs (*amyL* and *amyA*) was calculated using REST2009 software based on the three selected reference genes (as determined above in the section ‘Reference Genes During the Exponential Growth Phase of *L. plantarum* WCFS1’). The mRNA sample isolated from the strain *L. plantarum* S21 at OD_600_ ∼ 0.3 was used as a control for comparative analysis (Figure 6). After around 5 min of induction (counted as *t* = 0 h after induction), the expression levels of *amyL* and *amyA* genes can be measured in all recombinant strains. They reached their highest levels at 3 h after induction for all strains. Interestingly, the strains with the constructs based on the Lp_2145 signal peptide exhibited the highest mRNA levels for both amylases compared to the other strains and the expression levels of *amyL* and *amyA* genes reached a peak of 46- and 58-fold upregulation, respectively, at 3 h after induction in comparison with the control. At 3 h after induction of the expression of *amyL*, the mRNA level of the strain with the Lp_2145 signal peptide-containing construct was ∼3-fold higher than the strain with the native signal peptide SP_AmyL and ∼2-fold higher than the other strains (*p* < 0.05), while no significant difference in mRNA levels between the strains with the constructs containing the SP_AmyA, Lp_3050, and Lp_0373 signal peptides was observed (*p* > 0.05). On the other hand, at 3 h after induction of the expression of *amyA*, the strains with the constructs containing the SP_AmyA and Lp_3050 signal peptides showed similar mRNA level (*p* > 0.05), which was 1.5- and 2.6-fold lower than the strains with the constructs containing the Lp_0373 and Lp_2145 signal peptides (*p* < 0.05), respectively. After this point, the mRNA levels declined for both amylases in all the strains, of which the strain with the construct containing the native signal peptide SP_AmyL displayed the fastest decline of the transcript level. Furthermore, the strains with the Lp_3050 signal peptide-containing construct showed higher mRNA levels at 12 h after induction compared to the other strains in both cases of *amyL* and *amyA* (*p* < 0.05).

## Discussion

*Lactiplantibacillus plantarum* has been considered as a potential microorganism for recombinant protein production and secretion. However, the heterologous protein secretion capacity of *L. plantarum* has not yet met the needs of industrial production. One of the approaches to enhance the secretion capacity is to find the most optimal signal peptides for the target protein. By testing 76 signal peptides in *L. plantarum* WCFS1, Mathiesen et al. (2009) showed that the most efficient signal peptide for the secretion of NucA was Lp_3050, followed by Lp_2145, while Lp_0373 was reported to drive the secretion of NucA in a broad range of *Lactobacillus* species (Karlskås et al., 2014). Hence, we selected these signal peptides to improve the production and secretion of the α-amylase AmyL from *L. plantarum* S21.

Real-time PCR is a robust tool for gene expression studies due to its high sensitivity and specificity, however, to obtain accurate results for gene expression quantification, a proper set of reference genes serving as internal controls is required. The expression of the housekeeping genes can vary with the changes of environmental elements or experimental conditions. Several studies reported different combinations of reference genes, which could be used in *L. plantarum* strains (Marco et al., 2007; Fiocco et al., 2008; Duary et al., 2010; Wu et al., 2016; Lin et al., 2018), nevertheless these findings were not consistent due to the variations in selecting candidate reference genes, number of reference genes and experimental conditions. Hence, validation of reference genes is essential for an accurate RT-qPCR analysis. In our work, we measured the expression levels of α-amylase genes in *L. plantarum* WCFS1 at several time points in the exponential growth phase, where growth of the strains was affected by the reduction of both nutrients and pH, thus the housekeeping genes, which were *gmk*, *gyrA*, and *gapB*, with most stable expression under these conditions were selected as reference genes.

Even though the constructs contain identical transcription initiation and termination signals, RT-qPCR study of the α-amylase genes with various signal peptides revealed high variation in transcript levels. The strains with the Lp_2145 signal peptide-containing constructs showed the highest mRNA levels for both *amyL* and *amyA.* The mRNA levels reached their highest at 3 h after induction for all strains and after this point, the mRNA levels declined slowly for both amylases, which suggests that the transcription ceases around this point and the induction has stopped. It was previously reported that increasing initial concentration of the inducing peptide (IP-673) did not significantly affect the expression level of a recombinant protein in pH non-controlled fermentations using the pSIP expression system (Nguyen et al., 2015), thus increasing initial concentration or addition of inducing peptide would not lead to further induction. After reaching their peaks, the mRNA levels decreased with different rates depending on the signal peptides, which may indicate various stability of the transcripts. The strains with the constructs containing the Lp_2145 signal peptide gave significantly high production of both mRNA levels and protein synthesis, which suggests high compatibility of both α-amylase genes and the Lp_2145 signal peptide sequence in terms of transcription and translation. This “compatibility” apparently increased the mRNA stability thanks to its secondary structure properties. Studies showed that the differences in mRNA secondary structures led to differences in mRNA half-life because of their resistance to the degradosome in the cell (Carpousis, 2007; Cho, 2017; Vargas-Blanco and Shell, 2020). Besides, the difference in size of *amyL* (2625 bp) and the truncated form of *amyA* (1323 bp) might have caused the disparity in mRNA secondary structure, resulting in different expression levels between these two. Nevertheless, the exact conclusion for these assumptions is beyond this study. The effects of the signal peptides as well as of the target genes’ length on their mRNA stability should be further investigated. In addition to mRNA secondary structure, it is widely known that the secretion capacity of heterologous proteins can be impacted by other factors affecting the translational phase (e.g., codon usage, protein folding rate) and secretion stage [e.g., interaction between secretory machinery components and the precursor protein, efficiency of signal peptidase, and interaction between secretory protein and cell wall (Mathiesen et al., 2009)].

For both *amyA* and *amyL* expression, constructs with the Lp_2145 signal peptide surpassed the other ones regarding transcript levels and total enzymatic activities, but their secretion efficiencies were among the lowest. High transcript levels and intracellular α-amylase activities but low growth rates of the strains carrying Lp_2145 SP-based constructs suggest that the secretory machinery is overloaded, resulting in high rates of intracellular target protein folding and cellular stress as well as poor secretion efficiencies. In contrast, constructs based on the SP_AmyA signal peptide showed the highest secretion efficiencies of both AmyL and AmyA, as well as relatively high volumetric and specific activities of both AmyL and AmyA. The construct with the SP_AmyL signal peptide also displayed high secretion efficiency of AmyL, but low transcript levels and low protein yields as well as enzymatic activities were observed. In addition to Lp_2145, Lp_0373 appears to be an efficient non-cognate signal peptide for secretion of AmyL as well. High specific activities of AmyL and AmyA obtained with the constructs based on the Lp_0373 SP also suggests the compatibility of this signal peptide with lactobacillal α-amylases. Surprisingly, Lp_3050, which has been shown to be an effective SP in other studies (Mathiesen et al., 2009; Karlskås et al., 2014) showed the lowest capacity to secrete the α-amylases in this study. Although the mRNA levels of constructs with the Lp_3050 signal peptide were higher than those with the SP_AmyA signal peptide, their low growth rates and α-amylase specific activities suggest secretion stress. This might be due to high protein production that clogs the secretion machinery resulting in large amounts of misfolded proteins that stress the bacteria. Our results indicate that not only the secretion efficiency, but also the transcript level and translation efficiency are critical factors affecting the secretion of the α-amylases. This study shows that significantly higher expression and secretion of the α-amylase AmyL from *L. plantarum* S21 can be achieved in *L. plantarum* WCFS1 with heterologous signal peptides in comparison with the expression and secretion reported previously with its native signal peptide SP_AmyL (Kanpiengjai et al., 2015b). Finally, it is worth to mention that although *L. plantarum* WCFS1 also secretes α-amylases (Plaza-Vinuesa et al., 2019), there was no significant similarity found in both nucleotide and polypeptide sequences of *L. plantarum* WCFS1 in comparison with *L. plantarum* S21 or *L. amylovorus* NRRL B-4549 α-amylases when compared by BLAST^®^^6^ and we did not detect extracellular amylase activity with the wild-type *L. plantarum* WCFS1 under the conditions of cultivation used here. Therefore, the host’s transcriptome and α-amylases did not interfere with RT-qPCR assays and α-amylase activity measurements.

## Conclusion

This study shows that both efficient overexpression and secretion of the α-amylase AmyL from *L. plantarum* S21 can be achieved in *L. plantarum* WCFS1 when using several heterologous signal peptides. Lp_2145 and Lp_0373 are suitable heterologous signal peptides for the overexpression and secretion of AmyL, leading to a 6.2- and 4.5-fold increase in total volumetric activity, respectively, and a 5.4- and 4.3-fold increase in enzyme activity in extracellular fractions, respectively, in comparison to when the native AmyL signal peptide is used. Although no clear correlation between secretion efficiency and transcript levels in constructs containing different signal peptide sequences was found, this study revealed that the exchange of the signal peptide led to significant change in the mRNA level of the target gene.

## Data Availability Statement

The original contributions presented in the study are included in the manuscript/Supplementary Materials, further inquiries can be directed to the corresponding author.

## Author Contributions

THN conceived the idea. AMT performed the experiments and collected the data. AMT and THN analyzed the data and wrote the manuscript. KU, AK, CK, GM, and DH contributed to the discussion. All authors contributed to the article and approved the submitted version.

## Conflict of Interest

The authors declare that the research was conducted in the absence of any commercial or financial relationships that could be construed as a potential conflict of interest.

## Footnotes

1 www.cbs.dtu.dk/services/SignalP/

2 bioinfo.ut.ee/primer3

3 blast.ncbi.nlm.nih.gov

4 www.unafold.org

5 www.premierbiosoft.com/netprimer/

6 blast.ncbi.nlm.nih.gov/Blast.cgi
