# Supplementary material for: Efficient Secretion and Recombinant Production of a Lactobacillal α-amylase in Lactiplantibacillus plantarum WCFS1: Analysis and Comparison of the Secretion Using Different Signal Peptides
Source: Front Microbiol. 2021 Jun 14;12:689413. doi: 10.3389/fmicb.2021.689413 (PMC8236982; doi:10.3389/fmicb.2021.689413)
Supplement: Supplementary file 1 [file Data_Sheet_1.pdf]

Supplementary data

Figure S1. Growth profiles of the recombinant *L. plantarum* WCFS1 strains harboring AmyL (A) and AmyA (B) secretion plasmids with different signal peptides in 200 mL of MRS containing 5 μg/mL erythromycin at 37°C. Values given are the average values from at least two independent experiments and the error bars indicate the standard deviation.

Figure S2. SDS-PAGE analysis of cell-free supernatant and lysate of *L. plantarum* WCFS1 strains at 9 h after induction overexpressing the ~100 kDa α-amylase encoded by *amyL* (A) and the ~50 kDa α-amylase encoded by *amyA* (B). The arrows indicate the bands of α-amylases, M denotes the Precision protein ladder (Bio-Rad). Non-induced strains harbouring the plasmid pLp_spAmyA_AmyL and pLp_spAmyA_AmyA were representatives for non-induced conditions in (A) and (B), respectively.

Tables

Table S1. Primers used for cloning in this study

**Primers**	**Primer sequences (5′→3′)**	**Restriction site underlined**
AmyL_SalI_Fw	GGCGGAGTCGACGATAGTTATACGACATCAACTGATGAC	*Sal*I
AmyL_EcoRI_Rv	AGTAGTGAATTCTTACGAAGTGCTTGATGTGC	*Eco*RI
401_spAmyA_EcoRI_Fw	GATAAGAATTCGGTACCCCGGGTTCGAA	*Eco*RI
401_spAmyA_SalI_Rv	GATTAGTCGACACTAGCCGCTTGAGCAACTTGTTTAGA	*Sal*I

Table S2. Strains and plasmids used in this study

Strains and plasmids	Characteristics	References/Sources
**Strains**		
E. coli NEB5α	Host strain	New England Biolabs (Frankfurt am Main, Germany)
L. plantarum WCFS1	Host, protein expression strain	Kleerebezem *et al.* (2003)
L. plantarum S21	Native strain	Kanpiengjai *et al.* (2014)
**Plasmids**		
pSIP401	*spp*-based expression vector with inducible promoter P_{sppA}; Erm^R	Sørvig *et al.* (2003)
pSIP409	*spp*-based expression vector with inducible promoter P_{sppQ}, Erm^R	Sørvig *et al.* (2005)
pSIP401 derivatives		
pLp_2145s_AmyA	*amyA* fused to Lp_2145	Mathiesen *et al.* (2009)
pLp_3050s_AmyA	*amyA* fused to Lp_3050	Mathiesen *et al.* (2008)
pLp_0373s_AmyA	*amyA* fused to Lp_0373	Mathiesen *et al.* (2008)
pLp_spAmyA_AmyA	*amyA* with its native signal peptide (SP_AmyA)	Mathiesen *et al.* (2008)
pLp_2145s_AmyL	*amyL* fused to Lp_2145	This study
pLp_3050s_AmyL	*amyL* fused to Lp_3050	This study
pLp_0373s_AmyL	*amyL* fused to Lp_0373	This study
pLp_spAmyA_AmyL	*amyL* fused to signal peptide of AmyA (SP_AmyA)	This study
pSIP409 derivative		
pLp_AmyL7	*amyL* with native signal peptide (SP_AmyL)	Kanpiengjai *et al.* (2015b)

Table S3. Sequences of the signal peptides used in this study.

**Signal peptides**	**Amino acid sequences of the signal peptides** (including 2 amino acids downstream of cleavage site)[a]
SP_AmyA	MKKKKSFWLVSFLVIVASVFFISFGFSNHSKQVAQA↓AS
SP_AmyL	MKKKKSFWLVSFLVIVASVFFISFGLSNHSNQVAQA↓DS
Lp_2145	MKKINKLMILGMLVFGVTGATMINPEMTTAAHA↓SA
Lp_3050	MKKFNFKTMLLLVLASCVFGVVVNVTTSLGPQTAITAQA↓SK
Lp_0373	MYTENTGKHHRNGLPVWLLPLLVVISFWGVSQNIMVVDA↓SS

[a]The arrows ↓ represent the cleave sites of the signal peptides as determined by SignalP-5.0 (www.cbs.dtu.dk/services/SignalP/) (Armenteros *et al.*, 2019)
