# Supplementary figures and images for: Efficient Secretion and Recombinant Production of a Lactobacillal α-amylase in Lactiplantibacillus plantarum WCFS1: Analysis and Comparison of the Secretion Using Different Signal Peptides
Source: Front Microbiol. 2021 Jun 14;12:689413. doi: 10.3389/fmicb.2021.689413 (PMC8236982; doi:10.3389/fmicb.2021.689413)

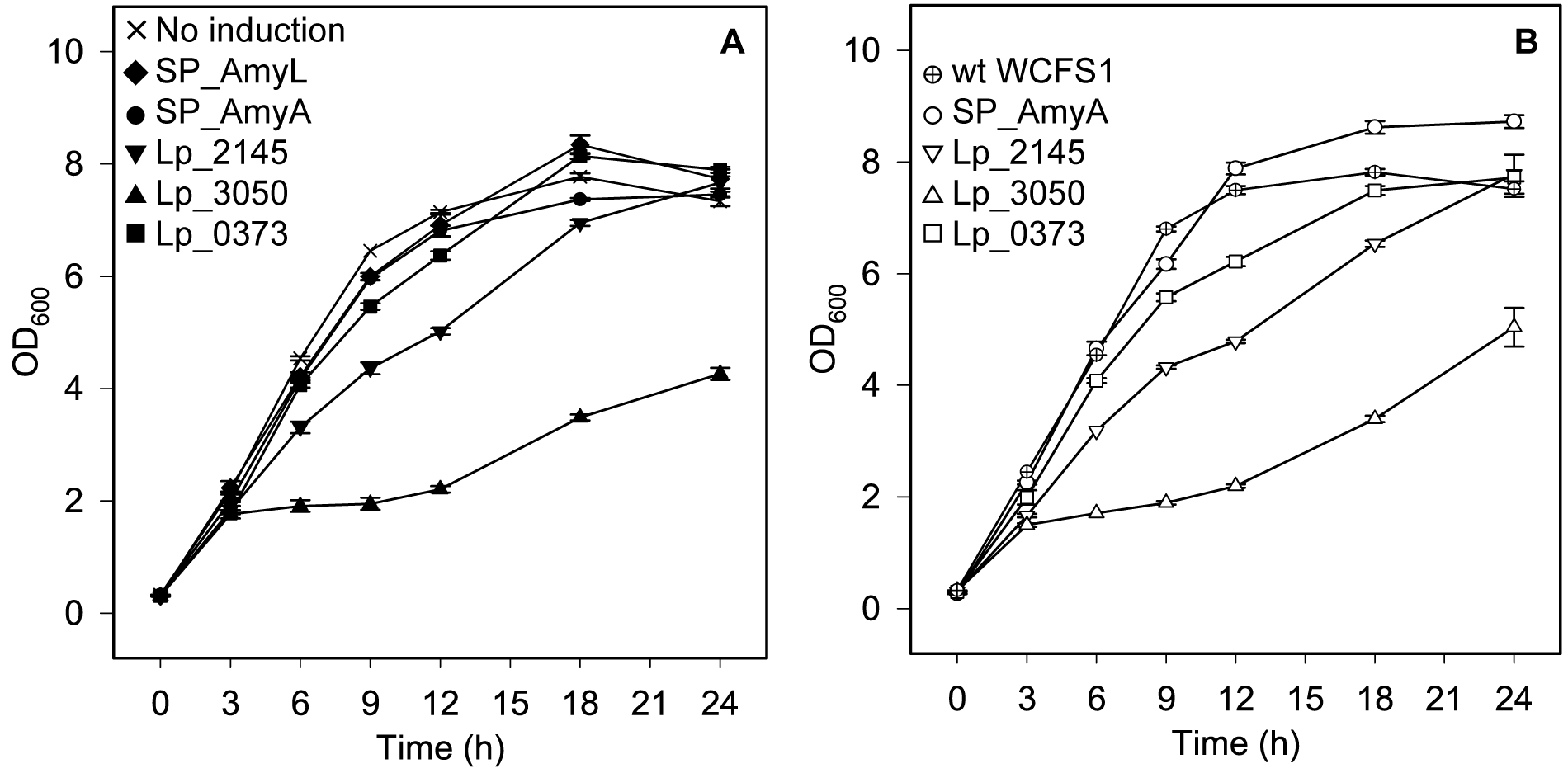

Supplement: Supplementary file 2 [file Image_1.TIF]

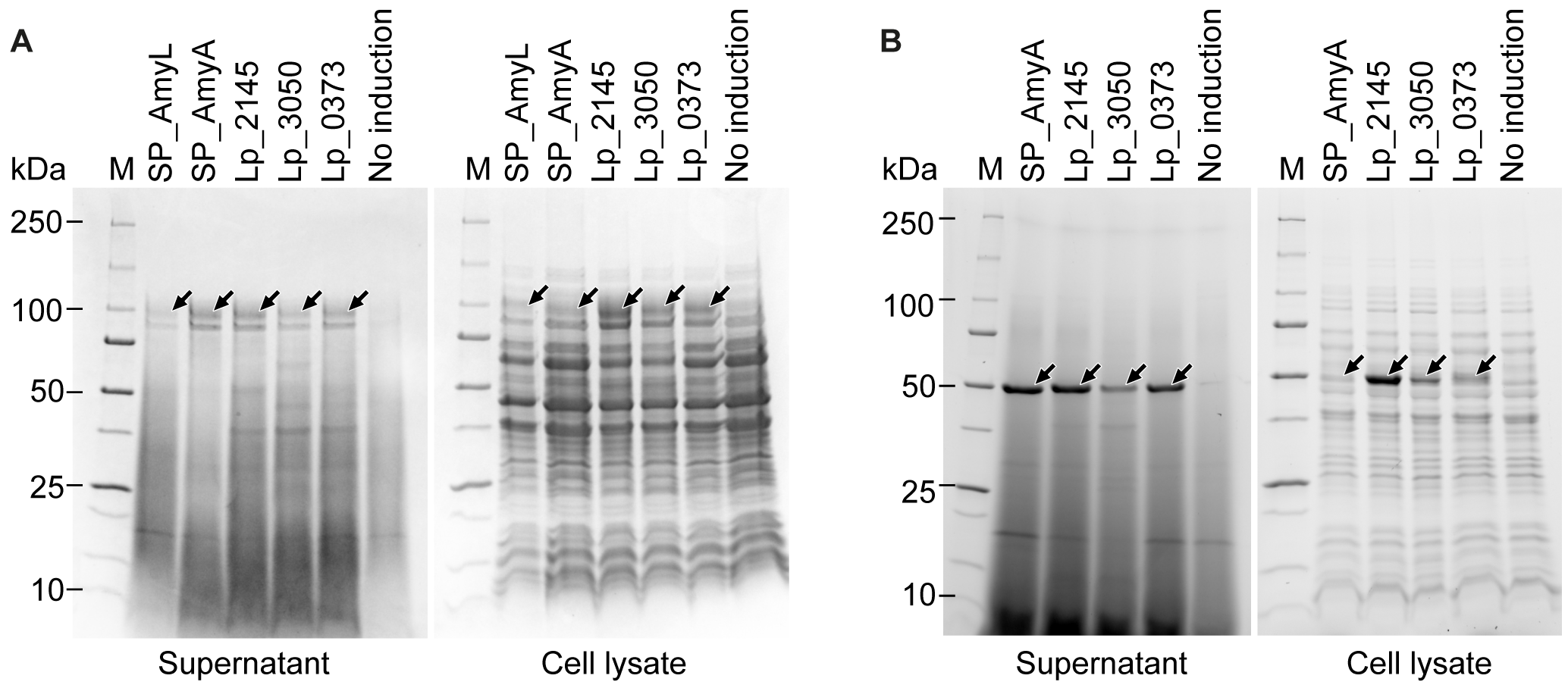

Supplement: Supplementary file 3 [file Image_2.TIF]
